# Das Konzept Gesundheitskompetenz im Wandel

**DOI:** 10.1007/s00103-024-04004-2

**Published:** 2025-01-15

**Authors:** Saskia Maria De Gani, Elena Alder, Anna-Sophia Beese

**Affiliations:** 1 Careum Stiftung, Zentrum für Gesundheitskompetenz, Pestalozzistrasse 3, 8032 Zürich, Schweiz; 2 Careum Hochschule Gesundheit, Teil der Kalaidos Fachhochschule, Gloriastrasse 18a, 8006 Zürich, Schweiz

**Keywords:** Patientenedukation, Polykrise, Infodemie, Befähigung, One Health, Patient education, Polycrisis, Infodemic, Empowerment, One Health

## Abstract

Gesundheitskompetenz ist eine zentrale Ressource für gesundheitsbezogene Entscheidungen. Seit den 1970er-Jahren veränderte sich das Verständnis des Begriffs jedoch auf vielfältige Weise. So wurde Gesundheitskompetenz zunächst lediglich unter dem Aspekt individueller Fähigkeiten betrachtet, in den letzten Jahrzehnten entwickelte sich ein stärker kontextabhängiges Verständnis. Trotz der Weiterentwicklung des Konzepts werden aktuelle gesellschaftliche und gesundheitliche Herausforderungen noch nicht ausreichend adressiert.

In diesem Beitrag werden die Veränderungen im Verständnis des Konzepts sowie die Bedeutung der Gesundheitskompetenz im Kontext der aktuellen Polykrise reflektiert. Dabei wird Bezug genommen auf ein von den Autorinnen erarbeitetes Konzeptpapier, das zeigt, dass Gesundheitskompetenz eine zentrale Ressource an der Schnittstelle zwischen Gesundheitsversorgung, Krankheitsprävention und Gesundheitsförderung darstellt, sich in unterschiedliche Ebenen unterteilen lässt und verschiedene Formen annimmt. Die Diskussion um Gesundheitskompetenz verdeutlicht, dass die Herausforderungen von Systemen, Organisationen und Lebenswelten berücksichtigt werden müssen, da in all diesen Kontexten Entscheidungen für die Gesundheit und das Wohlbefinden getroffen werden.

Gerade im Umgang mit den aktuellen gesellschaftlichen Herausforderungen nimmt die Bedeutung der Gesundheitskompetenz als zentrale Ressource noch zu und gewinnt als soziale Gesundheitsdeterminante an zusätzlicher Relevanz. Maßnahmen zur Stärkung der Gesundheitskompetenz können nur dann effektiv entwickelt, implementiert und evaluiert werden, wenn ein gemeinsames Verständnis von Gesundheitskompetenz besteht, aktuelle Herausforderungen und Kontexte berücksichtigt werden sowie bei der Maßnahmenumsetzung zentrale Akteur:innen gemeinsam agieren.

## Abstract (en)

Health literacy is a central resource for health-related decisions. Since the 1970s, however, the understanding of this term has changed in several ways. Initially, health literacy was considered only from the point of view of individual competencies, but in recent decades it has evolved into a more contextual understanding. Despite this conceptual evolution, current social and health challenges are still not adequately addressed.

This article reflects on the developments in the understanding of the concept and on the relevance of health literacy in the face of the current polycrisis. Reference is made to a recent concept paper developed by the authors, which shows that health literacy is a central resource at the interface of healthcare, disease prevention, and health promotion and that it can occur at different levels and in different forms. The discussion on health literacy also highlights the need to consider the challenges of systems, organizations, and settings, as decisions about health and well-being are made in all these contexts.

The importance of health literacy as a key resource is increasing, particularly in addressing current social challenges, and is gaining additional relevance as a social determinant of health. Health literacy interventions can only be effectively developed, implemented, and evaluated if a shared understanding of health literacy exists, current challenges and contexts are considered, and key stakeholders act together when implementing measures.

## Hintergrund

Gesundheitskompetenz ist eine zentrale Ressource, um mit Gesundheitsinformationen und -dienstleistungen umgehen und Entscheidungen für die Gesundheit und das Wohlbefinden treffen zu können [1]. Der Begriff Gesundheitskompetenz wurde seit seinem Ursprung in den 1970er-Jahren unterschiedlich definiert, stetig entwickelt und in verschiedenen Kontexten verbreitet [2]. Die jeweiligen Herausforderungen des Gesundheitssystems zur entsprechenden Zeit prägten dabei maßgeblich die Weiterentwicklung des Konzepts. Ein gemeinsamer Nenner dieser facettenreichen Perspektiven und Definitionen von Gesundheitskompetenz ist die Bedeutung des Konzepts für eine informierte Entscheidungsfindung, basierend auf dem adäquaten Umgang mit Gesundheitsinformationen und -diensten. Gesundheitskompetenz trägt diesem Verständnis entsprechend dazu bei, dass Menschen über die Kompetenzen verfügen, informierte Entscheidungen zu treffen, die sich positiv auf ihre Gesundheit und ihr Wohlbefinden auswirken, was wiederum das Gesundheitssystem entlastet [1, 3].

Der zentrale Beitrag von Gesundheitskompetenz für die individuelle Gesundheit sowie für die öffentliche Gesundheit und das Gesundheitssystem wird von verschiedenen Seiten, d. h. auch von relevanten Akteur:innen in der Forschung, Praxis und Policy, zunehmend anerkannt. Gleichzeitig besteht ein großer Förderbedarf: Europäische Studien zeigen, dass ein Großteil der Bevölkerung in Europa häufig über Schwierigkeiten im Umgang mit Gesundheitsinformationen berichtet und damit eine geringe Gesundheitskompetenz aufweist [4]. In der Schweiz und in Deutschland liegt der Anteil der Bevölkerung mit geringer Gesundheitskompetenz bei 49 % bzw. bei 59 % [5, 6]. Betrachtet man die 4 Schritte der Informationsverarbeitung (Informationen finden, verstehen, beurteilen, anwenden), zeigt sich, dass die Bevölkerung sowohl allgemeine Schwierigkeiten mit dem Finden und dem Verstehen als auch mit dem Beurteilen und dem Anwenden von Gesundheitsinformationen hat [5, 6]. Gerade das Beurteilen der Zuverlässigkeit der Informationen und ihrer Quellen sowie das Unterscheiden von zuverlässigen Informationen und Fehl- oder Falschinformationen stellen die Bevölkerung vor große Schwierigkeiten. Darüber hinaus fällt es vielen Menschen schwer, mit digitalen Informationen im Internet angemessen umzugehen oder sich in den oftmals komplexen Wirren des Gesundheitssystems orientieren zu können. Die digitale und die Navigations-Gesundheitskompetenz^1^ der Bevölkerung in der Schweiz und Deutschland fallen entsprechend im Vergleich zur generellen Gesundheitskompetenz noch geringer aus: 72 % (CH) und 76 % (DE) bzw. 74 % (CH) und 83 % (DE) der Befragten berichten von häufigen Schwierigkeiten im Umgang mit digitalen Gesundheitsinformationen oder mit Informationen zur Orientierung im Gesundheitssystem [5–7].

Die Datenlage zeigt eindeutig, dass es wichtig und dringend ist, die Gesundheitskompetenz der Bevölkerung und insbesondere von spezifischen Bevölkerungsgruppen (z. B. älteren Menschen, Menschen mit geringer sozialer Unterstützung oder in prekärer finanzieller Lage) zu stärken. Bisher wurden jedoch gerade auf politischer Ebene nur in einzelnen Ländern systematische Maßnahmen zur Stärkung der Gesundheitskompetenz ergriffen, wie auch folgendes Beispiel aus der Schweiz verdeutlicht: So ist die Stärkung der Gesundheitskompetenz in der Gesundheitsstrategie 2030 des Schweizer Bundesrates zwar offiziell als eines von 8 Zielen prominent verankert [8], jedoch wird dieses Ziel auf nationaler Ebene bisher ohne konkrete Umsetzungsschritte und wenig koordiniert verfolgt. Gerade die zentrale Koordination und konkrete Maßnahmenplanung wären jedoch relevant für die Stärkung der Gesundheitskompetenz [9].

Trotz des bereits seit mehreren Jahren bekannten Bedarfs zur Förderung der Gesundheitskompetenz der Bevölkerung sind deren Dringlichkeit und Relevanz in den letzten Jahren weiter gestiegen. Seit geraumer Zeit werden unsere Gesundheit, unser Wohlbefinden sowie unsere Gesellschaft vermehrt von rasch aufeinander folgenden, komplexen und disruptiven Dynamiken herausgefordert. Ein wesentlicher Treiber dieser Dynamiken ist die sogenannte Polykrise, in der wir uns aktuell befinden [10], d. h. eine Kumulation globaler Krisen, die sich gegenseitig beeinflussen und verstärken, sodass die Gesamtwirkung die Summe der einzelnen Krisen übersteigt [11].

Eine dieser Herausforderungen stellt die rasch voranschreitende digitale Transformation und die damit verbundene wachsende Anzahl digitaler Gesundheitsdaten, -informationen und -services dar. Diese Entwicklung geht mit der sogenannten Infodemie einher, einem Überfluss an vertrauenswürdigen, aber auch Falsch- und Fehlinformationen, welcher etliche Stolperfallen in Bezug auf informierte Entscheidungsprozesse birgt [12]. Mit Blick auf die rasanten Entwicklungen der digitalen Technologien, insbesondere der künstlichen Intelligenz, wird die Menge an Falsch- oder Fehlinformationen (als Text, Bilder oder Videos) voraussichtlich weiter zunehmen. Aus diesem Grund wird die Infodemie vom World Economic Forum als eine der größten aktuellen und zukünftigen Risiken betrachtet [13].

Eine weitere große gesundheitliche und gesellschaftliche Herausforderung ist der Klimawandel bzw. die Klimakrise. Diese Entwicklung hat verheerende Folgen für die Gesundheit und das Wohlbefinden von uns allen. Dazu gehören u. a. eine rasche Zunahme der Umweltmigration und die Verbreitung von bislang unbekannten Infektionskrankheiten aufgrund der langfristigen Zerstörung entscheidender Ökosysteme [14]. Einige weitere große Herausforderungen unserer Gesellschaft liegen im Gesundheitssystem (z. B. Fachkräftemangel, Ineffizienzen, Fehlanreize, steigende Kosten) sowie in der Zunahme gesundheitlicher und sozialer Ungerechtigkeiten [15].

Diese Ausführungen zeigen bereits, dass die allgemeine Bevölkerung, aber gerade auch (Gesundheits)fachpersonen spezifische Kompetenzen benötigen, um inmitten dieser Polykrise entscheidungs- und handlungsfähig zu bleiben. Der Bedarf an Gesundheitskompetenz wird also noch größer, damit Individuen sich heute und zukünftig im Dschungel von Informationen und Dienstleistungen zurechtfinden und informierte Entscheidungen für die Gesundheit und das Wohlbefinden von sich selbst und von anderen treffen können.

Das Konzept der Gesundheitskompetenz hat sich über die Jahre – ausgehend von einem individuumbezogenen hin zu einem relationalen Verständnis – stetig weiterentwickelt, was mitunter zu unterschiedlichen Verständnissen und Definitionen geführt hat [2, 16, 17]. Trotz dieser Weiterentwicklungen berücksichtigten die bisherigen Konzepte und Definitionen von Gesundheitskompetenz die aktuellen Herausforderungen, die mit der Polykrise einhergehen, sowie ihre Bedeutung und Auswirkungen auf die gesundheitsbezogenen Entscheidungen nur unzureichend [18, 19]. Ziel dieses Beitrags ist es, die Rolle der Gesundheitskompetenz in der aktuellen Polykrise zu reflektieren und ein neu eingeführtes Konzeptverständnis von Gesundheitskompetenz [18, 19] zu diskutieren. Dabei wird ausgehend von einer Übersicht über bisherige Konzeptverständnisse deren Transformation im Laufe der Zeit analysiert. Anschließend wird eine Weiterentwicklung des Konzeptes beschrieben sowie mögliche Implikationen für das öffentliche Gesundheitswesen und die Gesundheitspolitik aufgezeigt.

## Die Evolution der Definitionen und Konzepte von Gesundheitskompetenz

In den letzten Jahren hat sich der Begriff „Gesundheitskompetenz“ im deutschsprachigen Raum zunehmend verbreitet, die ersten Definitionen und Konzepte stammen jedoch aus dem angloamerikanischen Raum, wo der Begriff „Health Literacy“ geprägt wurde. Der konzeptionelle Ursprung von Health Literacy liegt in den Erziehungswissenschaften der 1970er-Jahre in den Vereinigten Staaten von Amerika [2, 20]. Seither hat der Begriff verschiedene Formen und Dimensionen angenommen und sich international und in anderen Fachgebieten, insbesondere in der Gesundheitsversorgung, verbreitet [20]. Trotz bzw. aufgrund der unterschiedlichen Verbreitung bestehen mittlerweile über 250 Definitionen von Gesundheitskompetenz [21]. Dies führt mitunter zu Missverständnissen, Unklarheiten und Konfusion sowohl in Forschung und Praxis als auch auf politischer Ebene [20], die der Etablierung des Konzepts sowie der systematischen Stärkung von Gesundheitskompetenz wenig zuträglich sind.

### Konzept an der Schnittstelle zwischen Gesundheitsversorgung, Krankheitsprävention und Gesundheitsförderung

Anfänglich wurde Gesundheitskompetenz als eine Ressource von Patient:innen verstanden, mit Herausforderungen im Bereich der medizinischen Versorgung angemessen umgehen zu können [22]. Somit lag der Fokus der ersten Konzeptverständnisse und der entsprechenden Maßnahmen zur Stärkung der Gesundheitskompetenz im Bereich der Gesundheitsversorgung und speziell bei der Patientenedukation und -schulung [23]. Darauf basierend wurde die Gesundheitskompetenz später zusätzlich als zentrale Ressource von Patient:innen identifiziert, mit nichtübertragbaren Krankheiten und diesbezüglichen Risikofaktoren umgehen zu können [24]. Das Konzept Gesundheitskompetenz wurde dabei verstärkt mit dem Konzept des Selbstmanagements, d. h. der individuellen Fähigkeit von Menschen, mit lang andauernden Krankheiten und den damit verbundenen täglichen Herausforderungen adäquat umgehen zu können [25], verknüpft. Das Verständnis von Gesundheitskompetenz wurde damit nicht nur als zentrale Ressource in der Gesundheitsversorgung verstanden, sondern schloss nun auch den Bereich der Krankheitsprävention ein. Das steigende Interesse am Thema, die vermehrte Anerkennung der Bedeutung sowie die Aufnahme in die Ottawa-Charta der Weltgesundheitsorganisation (WHO) im Jahr 1986 [26] haben schließlich dazu geführt, dass das Konzept der Gesundheitskompetenz auch den Bereich der Gesundheitsförderung adressiert.

Wegweisend definiert wurde Gesundheitskompetenz von der WHO im Jahr 1998 in ihrem Glossar zur Gesundheitsförderung als „kognitive und soziale Fähigkeiten, die die Motivation und die Fähigkeit des Einzelnen bestimmen, sich Zugang zu Informationen zu verschaffen, sie zu verstehen und so zu nutzen, dass Gesundheit gefördert und erhalten wird“ [27]. Darauf aufbauend wurde Gesundheitskompetenz im Laufe der Zeit als das Wissen, die Fähigkeiten und die Motivation verstanden, um gesundheitsbezogene Informationen zu verstehen und zu nutzen – und dadurch gesunde Entscheidungen für das Leben zu treffen, sich präventiv und gesundheitsförderlich zu verhalten und aktiv an der medizinischen Versorgung teilzunehmen [2]. Seither bildet Gesundheitskompetenz eine zentrale Schnittstelle zwischen den 3 Bereichen und den Konzepten der Gesundheitsversorgung, Krankheitsprävention und Gesundheitsförderung. Gesundheitskompetenz begrenzt sich jedoch nicht nur auf das Gesundheitswesen, sondern schließt andere Sektoren wie das Bildungs- und Sozialwesen, die Technologie- und Kommunikationsbranche sowie weitere Bereiche, die ebenfalls einen großen Einfluss auf Gesundheit und Wohlbefinden haben, mit ein [3].

### Ebenen der Gesundheitskompetenz

Im Jahr 2000 wurde ein weiteres Konzeptverständnis eingeführt, das 3 aufeinander aufbauende Ebenen umfasst [28]: Die „funktionale Gesundheitskompetenz“ beinhaltet die Grundkompetenzen, vor allem Lese‑, Schreib- und Rechenkompetenzen, um Gesundheitsinformationen im Alltag zu verstehen. Die ersten Definitionen von Gesundheitskompetenz aus dem angloamerikanischen Raum bezogen sich hauptsächlich auf diese Ebene. Aufbauend auf diesen Grundkompetenzen folgt die sogenannte interaktive Gesundheitskompetenz, welche erweiterte kognitive und soziale Kompetenzen umfasst, die die Nutzung von Gesundheitsinformationen in Interaktion mit dem Gesundheitssystem ermöglichen. Schließlich folgt die höchste Stufe, die kritische Gesundheitskompetenz, welche fortgeschrittene Kompetenzen umfasst, die es u. a. ermöglichen, eine kritische Bewertung von Gesundheitsinformationen vorzunehmen [28]. Im Laufe der Zeit wurde unter der kritischen Gesundheitskompetenz zunehmend auch die Fähigkeit verstanden, über verschiedenste Determinanten der Gesundheit zu reflektieren und daraus aktiv Entscheidungen abzuleiten [29]. Dadurch wurde auch eine Verbindung zum Empowerment-Ansatz geschaffen, der davon ausgeht, dass Gesundheitskompetenz Menschen in mehrfacher Hinsicht dazu befähigt, Verantwortung und Gestaltungswillen im eigenen Gesundheits- und Versorgungsprozess zu übernehmen [30].

### Vom individuumbezogenen zum relationalen Verständnis

Die ersten Definitionen und Konzeptverständnisse von Gesundheitskompetenz, inklusive derjenigen von Nutbeam (2000; [28]), sahen die Verantwortung für gesundheitsbezogene Entscheidungen eher beim Individuum, weshalb sich die Maßnahmen hauptsächlich auf Verhaltensänderungen des Einzelnen konzentriert haben [31]. Aktuellere Konzepte von Gesundheitskompetenz begannen jedoch zu betonen, dass gesundheitsrelevante Entscheidungen in Interaktionen mit anderen Menschen und mit dem System getroffen werden und dass Gesundheitskompetenz durch soziale und umweltbezogene Aspekte sowie den Kontext und die Lebenswelten der Menschen beeinflusst wird [31]. Die Definition von Sørensen et al. (2012), die sich anfänglich besonders im europäischen Raum stark verbreitet hat und die Grundlage für die erste europäische Erhebung der Gesundheitskompetenz in der Bevölkerung bildete (European Health Literacy Survey (HLS) des „Action Network on Measuring Population and Organizational Health Literacy“ (M-POHL); [4]), hebt diese Komponenten der Gesundheitskompetenz hervor und stellt Gesundheitskompetenz als soziale Determinante von Gesundheit in den Vordergrund. Die Definition und das Modell von Sørensen et al. (2012) berücksichtigen dabei sowohl die 3 Bereiche Gesundheitsversorgung, Krankheitsprävention und Gesundheitsförderung als auch die 4 Informationsschritte, die im Umgang mit Gesundheitsinformationen relevant sind: das Finden, das Verstehen, das Beurteilen und das Anwenden von Informationen [1].

Obwohl der individuelle Aspekt nach wie vor relevant geblieben ist, d. h., Gesundheitskompetenz basiert auf den persönlichen Kompetenzen jedes einzelnen Menschen, rückte der relationale und soziale Charakter von Gesundheitskompetenz mit der Zeit immer mehr in den Fokus der Diskurse zur Gesundheitskompetenz. Folglich ist es für ein ganzheitliches und aktuelles Verständnis von Gesundheitskompetenz relevant, dass die Herausforderungen und die Komplexität von Systemen, Organisationen und Lebenswelten berücksichtigt werden, weil sie die Kontexte darstellen, in denen Gesundheitskompetenz entsteht und gesundheitsbezogene Entscheidungen getroffen werden [32].

### Formen der Gesundheitskompetenz

Die gesellschaftlichen Entwicklungen in den letzten Jahren, wie beispielsweise die digitale Transformation, haben dazu geführt, dass Gesundheitsinformationen und -services vermehrt digital, im Internet und in den sozialen Medien, generiert und verbreitet und auch über diese Kanäle genutzt werden. Dies hat zur Einführung des Begriffs der digitalen Gesundheitskompetenz geführt, die zentral ist für den Umgang mit digitalen Gesundheitsinformationen und -angeboten [33]. Eine weitere Entwicklung betrifft die Gesundheitssysteme selbst. Sie sind in den vergangenen Jahren aus verschiedenen Gründen immer komplexer geworden, u. a. durch die vergrößerte Auswahl von Behandlungsoptionen und die verstärkte Spezialisierung der Medizin. In diesem Zusammenhang wurde die „Navigations-“ bzw. „navigationale Gesundheitskompetenz“ eingeführt, eine Voraussetzung, um sich im Gesundheitssystem orientieren und die am besten geeignete Gesundheitsversorgung für sich oder für die Gesundheit von anderen wählen zu können [34]. In ähnlicher Weise wurden in den letzten Jahren weitere Formen von Gesundheitskompetenz, beispielweise „impfbezogene Gesundheitskompetenz“ [35] oder „mentale Gesundheitskompetenz“ [36], definiert.

### Ein gemeinsames Verständnis von Gesundheitskompetenz für den Schweizer Kontext

Aufgrund der oben beschriebenen Entwicklungen, der aktuellen Herausforderungen und der unterschiedlichen Verständnisse des Konzepts Gesundheitskompetenz wurde in der Schweiz als Reaktion auf die Ergebnisse des Health-Literacy-Surveys 2019–2021 [6] versucht, ein gemeinsames Verständnis für Gesundheitskompetenz zu schaffen [18, 19]. Dieser Entwicklungsprozess fand im Auftrag des Bundesamtes für Gesundheit, in Kooperation mit der Allianz Gesundheitskompetenz und unter Leitung der Autorinnen dieses Artikels des Careum Zentrums für Gesundheitskompetenz statt. Erst ein gemeinsames Verständnis erlaubt es, koordinierte Strategien und Maßnahmen zur Stärkung der Gesundheitskompetenz zu lancieren. Dieser partizipative Prozess führte schließlich zu einer Arbeitsdefinition von Gesundheitskompetenz, die außerdem von der „Gesundheitskompetenz-Stärkung“ abgegrenzt wurde.

Nach dieser Arbeitsdefinition umfasst Gesundheitskompetenz „ein Bündel von Kompetenzen, um proaktiv mit gesundheitsbezogenen Informationen, Dienstleistungen und Herausforderungen umzugehen. Dadurch werden Menschen befähigt, sich um die Gesundheit und das Wohlbefinden von sich und anderen zu kümmern“ [18, S. 17]. Ausgehend vom relationalen und kontextspezifischen Verständnis von Gesundheitskompetenz wurde der Begriff der „Gesundheitskompetenz-Stärkung“ definiert. Diese umfasst alle „Praktiken, Prozesse, Strukturen und Strategien unterschiedlicher Akteur:innen, innerhalb institutioneller, sektoraler oder regionaler Grenzen und darüber hinaus, durch die Menschen befähigt werden, ihre Gesundheitskompetenz zu entwickeln und zu stärken“ [18, S. 17]. Dieses Verständnis von Gesundheitskompetenz greift damit weiter und schließt nicht nur den Umgang mit gesundheitsrelevanten Informationen und Dienstleistungen ein, sondern insbesondere auch den Umgang mit aktuellen Herausforderungen, mit denen wir u. a. durch die Polykrise konfrontiert sind. Weiter umfasst dieses Verständnis verschiedene Perspektiven, Ebenen und Themenbereiche. Dies ist zentral, um nachhaltige, breit abgestützte und koordinierte Maßnahmen und Strategien zur Stärkung der Gesundheitskompetenz zu entwickeln [9].

## Gesundheitskompetenz als zentrale Ressource unserer Gesellschaft im Umgang mit der Polykrise

Gesundheitskompetenz kann als Mediator- und Moderatorvariable unterschiedlicher Gesundheitsdeterminanten, wie etwa der sozioökonomischen, der umweltbezogenen oder auch der kommerziellen und digitalen Determinanten der Gesundheit, betrachtet werden [37, 38]. Andererseits stellt die Gesundheitskompetenz selbst eine wichtige soziale Gesundheitsdeterminante dar [39] und ist gemäß mehreren Studien ein relevanter Prädiktor für den (selbstberichteten) Gesundheitsstatus [38, 40, 41]. Über die Stärkung der Gesundheitskompetenz insbesondere auf institutioneller und systemischer Ebene kann deshalb nicht nur ein wichtiger Beitrag zur Gesundheit von Individuen und der öffentlichen Gesundheit, sondern auch zur Schaffung von mehr gesundheitlicher und sozialer Gerechtigkeit geleistet werden. Dies trifft speziell auf vulnerable Gruppen wie Menschen mit chronischen Erkrankungen, mit finanziellen Problemen oder wenig sozialer Unterstützung zu [18, 37].

Gesundheitskompetenz und ihre Stärkung sind in der heutigen Zeit und wohl auch zukünftig von enormer Relevanz für selbstbestimmte Entscheidungen und Handlungen, die zur Gesundheit und zum Wohlbefinden von uns Menschen beitragen. Mit Blick auf die Klimakrise und die starke Belastung der Ökosysteme wird jedoch klar, dass wir gleichzeitig auch die Gesundheit und das Wohlbefinden anderer Menschen, der Tiere und der Ökosysteme mitdenken müssen. Das bedeutet, dass wir in Anbetracht der zahlreichen voneinander abhängigen Determinanten zusätzliche Kompetenzen benötigen, um fundierte Entscheidungen für das tägliche Leben treffen zu können. Die komplexen Wechselwirkungen werden im sogenannten One-Health-Ansatz zusammengefasst. Dieser integrierte und von der WHO forcierte Ansatz unterstreicht, dass die Gesundheit und das Wohlergehen von Menschen, Haus- und Wildtieren, Pflanzen sowie der weiteren Umwelt eng miteinander verbunden und voneinander abhängig sind [42]. Entsprechend benötigen Individuen, Fachpersonen, Entscheidungsträger:innen und Organisationen innerhalb und außerhalb des Gesundheitswesens eine Art „One Health Literacy“, verstanden als das Wissen, die Motivation und die Fähigkeiten von Menschen, relevante Informationen zu finden, zu verstehen, zu beurteilen und anzuwenden, um im Alltag Entscheidungen treffen zu können, die dazu beitragen, die Gesundheit und die Lebensqualität von uns Menschen, den Tieren und der Umwelt nachhaltig und in einem ausgewogenen Verhältnis zu fördern ([43], eigene Übersetzung).

Aus einer systemischen Perspektive kann Gesundheitskompetenz auch in der Bewältigung der aktuellen Herausforderungen des Gesundheitssystems wie dem Fachkräftemangel, den Qualitäts- und Effizienzproblemen sowie den steigenden Gesundheitskosten einen Beitrag leisten. So haben Studien in Europa gezeigt, dass eine geringe Gesundheitskompetenz u. a. im Zusammenhang mit einer vermehrten Inanspruchnahme von Gesundheitsleistungen steht [5, 6, 44]. Über die Gesundheitskompetenz-Stärkung auf institutioneller und systemischer Ebene besteht folglich das Potenzial, die Versorgungsqualität zu steigern, Fachkräfte zu entlasten und Kosten zu reduzieren.

## Implikationen für das Gesundheitswesen und die Gesundheitspolitik

Damit Menschen das nötige Wissen, die Motivation und die Fähigkeiten erwerben und entwickeln können, um mit Herausforderungen im Zusammenhang mit der Gesundheit und dem Wohlbefinden umgehen zu können, ist es dringend erforderlich, die Gesundheitskompetenz auf verschiedenen Ebenen (individuell, gesellschaftlich, organisational, strukturell) zu stärken. Hierfür ist die koordinierte Zusammenarbeit unterschiedlicher Akteur:innen und Disziplinen essenziell. Dies bedingt u. a. auch die Partizipation der Zielgruppen. Dabei gilt es insbesondere, benachteiligte und vulnerable Bevölkerungsgruppen einzubinden und sie dabei zu unterstützen, sich zukünftig an relevanten Entscheidungen zu ihrer Gesundheit und ihrem Wohlbefinden zu beteiligen. Damit kann entsprechend die gesundheitliche Chancengerechtigkeit erhöht werden [3, 37].

Bisherige Aktivitäten zur Stärkung der Gesundheitskompetenz fokussierten häufig auf den Bereich der Gesundheitsversorgung. Gerade aber die Prävention und die Gesundheitsförderung bieten großes Potenzial für die öffentliche Gesundheit bzw. auch für die Optimierung der Gesundheit und des Wohlbefindens nicht nur von uns Menschen, sondern auch von Tieren und Ökosystemen (im Sinne des One-Health-Ansatzes). Deshalb gilt es, politische Strategien und Maßnahmen nicht nur in der Human- und Veterinärmedizin neu zu denken, sondern beispielweise auch im Bildungs- und Sozialbereich, im Verkehrs- und Umweltsektor, und zwar sowohl auf internationaler, nationaler als auch kantonaler bzw. Länder- und Gemeindeebene [3]. Ein gemeinsames Verständnis von Gesundheitskompetenz bildet dafür eine wichtige Grundlage. Auf dieser Basis können Aktivitäten und Maßnahmen zentraler Akteur:innen koordiniert und damit nationale Aktionspläne und zielgerichtete Strategien zur Stärkung der Gesundheitskompetenz entwickelt und umgesetzt werden [9, 18].

Dies kann dann wiederum beispielsweise mithilfe des *Public Health Action Cycle*, einem Modell zur Entwicklung, Implementierung und Evaluation von Gesundheitsinterventionen, erfolgen [9, 45]. Das Modell beinhaltet 5 verschiedene Phasen zur Entwicklung, Umsetzung und Evaluation von politischen Gesundheitsstrategien [9]. Die erste Phase dieses Modelles beinhaltet die Analyse der Situation bzw. des Problems und würde in diesem Fall eine Erhebung der Gesundheitskompetenz auf den Ebenen der Bevölkerung, von Fachpersonen und von Organisationen und Systemen beinhalten [9]. In der Situationsanalyse ist eine gemeinsame Definition von Gesundheitskompetenz zentral, damit auch genau dies untersucht und passende Erhebungsinstrumente verwendet werden können. Auch beim Agenda-Setting (2. Phase) und bei der Entwicklung von politischen Strategien (3. Phase) ist ein klares Verständnis von Gesundheitskompetenz entscheidend, insbesondere für die Etablierung von Netzwerken, Allianzen und Koalitionen mit den zentralen Akteur:innen aus der Forschung, Politik und Praxis. Dies beinhaltet auch die Partizipation der Bevölkerung, gerade wenn ein One-Health-Ansatz verfolgt werden soll [43]. Damit sich die Bevölkerung an Policy-Prozessen und Entscheidungen beteiligen und einen aktiven Beitrag leisten kann, benötigt sie jedoch wiederum gewisse Kompetenzen und Möglichkeiten [9]. Die Polykrise stellt uns nicht nur vor Herausforderungen, sondern bietet gleichzeitig auch Chancen, sogenannte „Windows of Opportunities“ [9], um die Gesundheitskompetenz der Bevölkerung durch die Zusammenarbeit verschiedener Akteur:innen auf unterschiedlichen Ebenen und die Entwicklung passender, koordinierter Prozesse, insbesondere auch politischer Strategien und Maßnahmen, nachhaltig zu stärken.

Für die erfolgreiche Implementierung dieser Prozesse, Strategien und Maßnahmen (4. Phase des Public Health Action Cycle) ist es jedoch wichtig, dass die notwendigen Kapazitäten aufgebaut (Capacity Building) und die dafür erforderlichen finanziellen und personellen Ressourcen bereitgestellt werden [9]. Hierfür ist ein gemeinsames Verständnis von Gesundheitskompetenz erneut relevant, damit die Tätigkeiten und Finanzierungen zielführend koordiniert werden können. Die 5. und letzte Phase beinhaltet die Evaluation, um zu prüfen, ob die Ziele auch tatsächlich erreicht und Wirkung erzielt wurde. Dazu gehört auch das regelmäßige Monitoring der Gesundheitskompetenz der Bevölkerung, von Gesundheitsfachpersonen, von Organisationen etc. [9]. Für ein effektives Monitoring, das es erlaubt, geplante Veränderungen zu beurteilen, ist es erneut zentral, ein gemeinsames Verständnis von Gesundheitskompetenz als Grundlage zu haben, das aktuelle Dynamiken berücksichtigt, weil sie einen Einfluss auf die wahrgenommenen Schwierigkeiten im Umgang mit Gesundheitsinformationen, -dienstleistungen und -herausforderungen haben können.

## Fazit

Die aktuelle Polykrise fordert und prägt unsere Gesellschaft und das Gesundheitssystem in verschiedenster Weise. Entsprechend sind wir Menschen auf spezifische Kompetenzen im Umgang mit gesundheitsrelevanten Informationen, Dienstleistungen und Herausforderungen angewiesen, damit wir gute und nachhaltige Entscheidungen für die Gesundheit und das Wohlbefinden sowohl von uns selbst als auch von anderen Menschen, den Tieren und Ökosystemen treffen können. Diese Herausforderungen und Kompetenzen sollten in das Konzept Gesundheitskompetenz und die Diskussionen zum Thema verstärkt aufgenommen werden. Somit kann sich das Konzept, wie es seit der ersten Konzeptualisierung über die Zeit immer wieder geschehen ist, kontinuierlich weiterentwickeln. Parallel zur Entwicklung des Konzepts hat Gesundheitskompetenz in den letzten Jahren eine wachsende Aufmerksamkeit in der Forschung, der Praxis und der Politik erfahren. Mit der Polykrise wird die zentrale Rolle von Gesundheitskompetenz als soziale Gesundheitsdeterminante noch deutlicher. Damit Maßnahmen zur Stärkung der Gesundheitskompetenz der Bevölkerung, der Angehörigen der Gesundheitsberufe, der Organisationen [46, 47] und der Systeme nachhaltig und effektiv entwickelt, umgesetzt und evaluiert werden können, sind jedoch ein gemeinsames Verständnis von Gesundheitskompetenz sowie eine koordinierte Arbeit zwischen zentralen Akteur:innen, Entscheidungsträger:innen und Sektoren dringend erforderlich. Dies kann die Gesellschaft dabei unterstützen, die Herausforderungen der Polykrise besser zu bewältigen.

## Footnotes

1 Navigations-Gesundheitskompetenz bezieht sich auf die Fähigkeit, sich mithilfe von Informationen im Gesundheitssystem zurechtzufinden und die am besten geeignete Gesundheitsversorgung für sich oder für andere wählen zu können [34].
